# Protein Nanocarriers: Targeted Theranostics for Cancer Treatment and Imaging

**DOI:** 10.3390/cancers18050832

**Published:** 2026-03-04

**Authors:** Reyhan Dilsu Colpan, Neil R. Thomas, Lyudmila Turyanska, Tracey D. Bradshaw

**Affiliations:** 1 Biodiscovery Institute, School of Pharmacy, University of Nottingham, Nottingham NG7 2RD, UK; 2 Biodiscovery Institute, School of Chemistry, University of Nottingham, Nottingham NG7 2RD, UK; neil.thomas@nottingham.ac.uk; 3 Faculty of Engineering, University of Nottingham, Nottingham NG7 2RD, UK; lyudmila.turyanska@nottingham.ac.uk

**Keywords:** protein nanocarriers, cancer theranostics, targeted delivery, overexpressed receptors

## Abstract

Protein-based nanocarriers have gained considerable attention for targeted cancer theranostic applications due to their inherent biocompatibility, biodegradability, and facile functionalisation. In addition, some of their properties, such as self-assembling nature, low immunogenicity (if species matched), molecular recognition ability, and lack of persistence due to degradation into proteinogenic amino acids, make them highly suitable for oncology-related applications. Each protein-based nanocarrier exhibits unique physicochemical and biological properties. In this review, we summarise recent advances in targeted protein-based nanocarriers, including albumin, lipoproteins, ferritin, viral protein capsids, fibrin type proteins and silk proteins, emphasising receptor-specific targeting mechanisms, the integration of various imaging modalities along with their advantages and limitations, and the importance of employing advanced preclinical models for translational theranostic applications. This review also discusses the most recent and significant studies in the field, providing useful insights into future directions of protein-based nanocarriers for cancer theranostics.

## Simple Summary

Cancer treatment and imaging are still limited since many drugs and imaging agents can neither effectively nor selectively reach tumour tissues. Therefore, new strategies are needed to improve drug and imaging agent delivery and reduce side effects. This review focuses on targeted protein-based nanocarriers as innovative devices for cancer diagnosis and therapy (theranostics), capable of delivering drug(s) and imaging agent(s) simultaneously. We discuss overexpressed protein receptors in cancer cells that differ from normal tissue expression and can be exploited for targeted delivery. This review summarises recent preclinical studies using protein nanocarriers as targeted theranostic platforms to improve cancer treatment, reduce side effects, and enable non-invasive tracking of treatment progress. Overall, protein nanocarriers represent promising devices that combine imaging modalities and targeting strategies for more effective cancer diagnosis and therapy in the future.

## 1. Introduction

Targeted theranostics, which combine therapeutic and diagnostic modalities within a single platform, represent a transformative approach in personalised oncology. By integrating targeted therapy with diagnostic imaging, theranostic systems can enable real-time monitoring of therapeutic responses, optimisation of drug dosage and reduction of systemic toxicity [1]. A wide range of therapeutic cargos, including chemotherapeutic and immunotherapeutic drugs, as well as imaging agents for computed tomography (CT), magnetic resonance imaging (MRI), positron emission tomography (PET), single-photon emission computed tomography (SPECT), and near-infrared imaging (NIR), have been reported in targeted cancer theranostics [2,3,4]. Whereas fluorescence imaging agents offer high resolution, development of novel NIR imaging agents will advance depth penetration capabilities.

**Figure 1:**
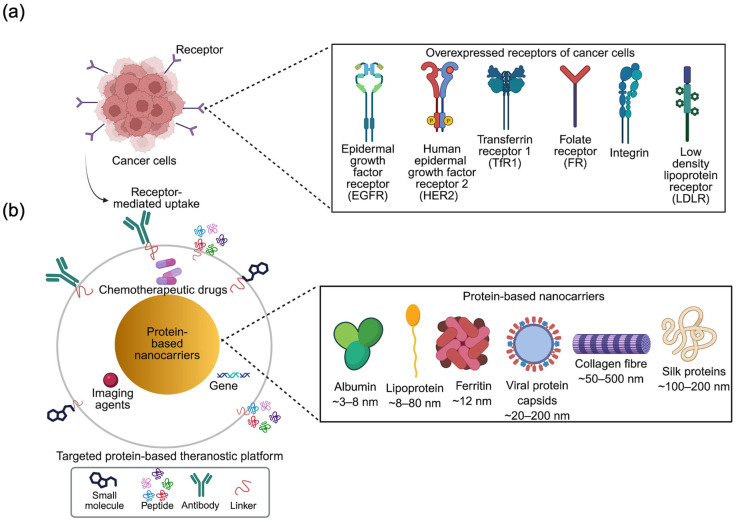
(**a**) Overexpressed receptors on cancer cells, including epidermal growth factor receptor (EGFR), human epidermal growth factor receptor 2 (HER2), transferrin receptor 1 (TfR1), folate receptor (FR), integrins, and low density lipoprotein receptor (LDLR), enable active targeting of protein-based nanocarriers for combined diagnostic and therapeutic (theranostic) delivery. (**b**) Protein-based nanocarriers for targeted cancer theranostics. Naturally derived (albumin, lipoprotein, ferritin, and collagen) and engineered protein assemblies (viral protein capsids and silk proteins) are used to deliver drugs and imaging agents to tumours via receptor-mediated active targeting using ligands such as small molecules, peptides, and antibodies. Created with BioRender.

Nanocarriers, such as liposomes [5], polymeric nanoparticles (NPs) [6], inorganic materials [7] and proteins, have been successfully used. Protein nanocapsules are considered advantageous because of their size, biocompatibility and biodegradability, receptor-mediated targeting ability, and multifunctional design opportunities with functionalisation for advanced theranostic applications [3]. This review focuses on recent research on protein-based nanocarriers for targeted cancer theranostics, highlighting their ability to target overexpressed receptors (Figure 1). Key examples of protein-based theranostic agents are discussed, with analysis of advanced preclinical studies, as well as current challenges and future perspectives.

## 2. Targeting Overexpressed Receptors

Targeting of the receptors overexpressed in tumour cells is a key strategy to achieve cancer selectivity and specificity [8]. Several receptors, overexpressed in cancers, are considered biomarkers, including the epidermal growth factor receptor (EGFR), human epidermal growth factor receptor 2 (HER2), transferrin receptor 1 (TfR1), folate receptor (FR), integrins, and low density lipoprotein receptor (LDLR; Figure 1a) [9].

EGFR is a transmembrane receptor tyrosine kinase (RTK) that is overexpressed in cancers, including glioblastoma (GBM) [10], breast [11], colorectal [12], and non-small cell lung cancer (NSCLC) [13]. EGFR mutations (e.g., EGFRvIII) and amplifications are prevalent in cancer, activating multiple cell signalling pathways (including RAS-RAF-MAPK and PI3K-AKT transduction), resulting in cell survival and uncontrolled proliferation [14]. Therefore, several targeted therapies such as erlotinib, gefitinib, and osimertinib have been used as first-line treatments to inhibit EGFR activity and block downstream pathways in the clinic [15]. HER2, a member of the EGFR family, plays a crucial role in tumourigenesis. Alterations in the *HER2* gene, including mutations, amplifications, or overexpression, are associated with the development of cancers [16], including breast [17], gastric [18], and colorectal [19] carcinomas, and HER2 upregulation has also been observed in ~80% of GBM tumours [20]. Previous studies have reported 20,000 HER2 receptors per cell in normal breast epithelial cells [21], whereas SKBR3 and AU565 breast cancer cells exhibit HER2 overexpression, with receptor numbers reaching 1–2 million per cell [22].

TfR1 (also known as CD71) is a type II transmembrane glycoprotein that plays a critical role in cellular iron uptake [23] via TfR1-mediated endocytosis—essential for deoxyribonucleic acid (DNA) synthesis and cell proliferation [24]. TfR1-mediated endocytosis delivers transferrin-bound iron (Fe^3+^) into cells, which is subsequently reduced to Fe^2+^ and utilised by ribonucleotide reductase to catalyse the conversion of ribonucleotides to deoxyribonucleotides, supporting DNA synthesis and cell proliferation [25]. TfR1 upregulation has been observed in many cancers to ensure sufficient intracellular iron ion availability to sustain their critical cellular processes [26]. Shen et al. reported key differences in TfR1 expression between normal and cancerous tissues, including those of the brain, ovary, colon, and lung [27]. For example, in GBM, TfR1 expression is enhanced in tumour cells compared to non-cancerous cells. It is also abundant on blood–brain barrier (BBB) endothelial cells, allowing transcytosis. Hence, TfR1 can serve as a multifunctional biomarker that integrates iron metabolism with oncogenic signalling, oxidative stress, and metabolic adaptation in cancer cells, making it a promising target for theranostic applications [28].

Folate, also known as vitamin B9, plays a unique role in tumourigenesis by acting as a non-toxic carrier of one-carbon units required for DNA and RNA synthesis, which are essential for cell division, DNA repair, cell signalling and methylation reactions [28,29]. The FR is overexpressed in many tumour types, facilitating both sustenance of nucleotide synthesis and storage phase of nucleotide pools [28]. FR isoform alpha (FRα) is overexpressed in head and neck cancers (>45%) and NSCLC (14–74%), highlighting its potential as a biomarker for targeted cancer theranostic approaches [29].

Integrins are transmembrane heterodimer receptors that play key roles in tumourigenesis, growth, invasion, metastasis, and drug resistance through interactions with extracellular matrix (ECM) and by regulating cell adhesion and mobility. They are classified into several groups, including Arg-Gly-Asp (RGD), collagen, laminin, and leukocyte-specific integrins [30]. Several studies have reported integrin upregulation in various cancers such as breast [31], lung [32], colorectal [33], and GBM [34]. Che et al. reported that integrins act as regulators of metabolic reprogramming (cancer hallmark; Warburg effect) in GBM [35]. LDLR is another transmembrane receptor that mediates cellular cholesterol uptake [9]. In cancers including breast cancer [36] and gliomas [37], it is frequently upregulated, and higher expression correlates with increased proliferation and poorer clinical outcomes.

Protein-based nanocarriers can offer inherent targeting capabilities or can be functionalised with specific receptor-targeting ligands to deliver the cargo to the tumour site, facilitating receptor-mediated uptake and accumulation of theranostic agents in cancer cells, thus improving both therapeutic outcome and imaging precision [28].

## 3. Protein-Based Nanocarriers

Protein-based nanocarriers are derived from natural or recombinant proteins and have gained significant attention owing to their biodegradability, biocompatibility, structural stability, amenability for surface modification, and reduced toxicity for targeted therapeutic, diagnostic, and theranostic applications [38]. They are recognised as safe substances, as they can be metabolised by digestive enzymes, are non-immunogenic and possess a uniform size [39,40]; additionally, their well-defined molecular protein structures allow precise and site-specific surface modification with targeting ligands, antibodies, and peptides through protein engineering [41]. Even without such modifications, these nanocarriers can achieve passive targeting via enhanced permeability and retention (EPR), accumulating in tumour tissues due to the porous vasculature and poor lymphatic drainage associated with the tumour microenvironment. Compared to polymeric or metallic nanoparticles, proteins can offer advantages, as mentioned above, which may enhance tumour accumulation—a consequence of the EPR effect. Protein-based nanocarriers provide versatility for encapsulation or conjugation of both hydrophilic and hydrophobic molecular drugs [42,43,44], siRNA [45,46], antigens [47,48], and imaging agents (organic dyes [49], quantum dots (QDs) [50,51]). There are several types of protein-based nanocarriers, including albumin, lipoprotein, ferritin, viral protein capsids, collagen fibre, and silk proteins that are currently considered promising for targeted cancer theranostics (Figure 1b).

Albumin is one of the most abundant circulating plasma proteins with a single chain (contains 585 amino acids) [52] that exists as ovalbumin (OVA), bovine serum albumin (BSA), and human serum albumin (HSA) [40]. Albumins possess a prolonged circulation time (≤19 days), a consequence of the ability of this innate circulating protein to bypass immune system detection. Albumin-based nanocarriers allow for improved targeted delivery to tumours via interaction with glycoprotein 60 (gp60, albondin) and secreted protein acidic and rich in cysteine (SPARC) proteins [53] and are considered promising nanocarriers for applications in cancer, diabetes, and infection [54]. Albumins can be formulated as NPs through desolvation (coacervation), emulsification, nanoparticle albumin-bound (nab) technology, and thermal treatment [55]. Abraxane, nanoparticle HSA-bound (Nab) paclitaxel (PTX) [55], was the first protein-based nanoformulation approved by the Food and Drug Administration (FDA) for the treatment of metastatic breast, pancreatic and non-small cell lung cancers [56]. The albumin-based nanocarrier stabilises pharmacokinetic properties of PTX, enabling personalised approaches by enhancing biodistribution and prolonging circulation time for improved therapeutic outcomes [54]. Manufacture is cost-effective and scalable, and also its size, distribution, and targeting abilities also support the feasibility of commercialisation and clinical translation.

Lipoproteins comprise lipid-protein complexes, such as high density lipoprotein, low density lipoprotein and very low density lipoprotein [39], with varying lipid-to-protein ratios [57]. They can be isolated from plasma or produced by mixing protein subunits and lipids [57]. These nanocarriers exploit receptor-mediated uptake by binding specific receptors, such as LDLR, scavenger receptor class B type 1 (SRB1), and adenosine triphosphate-binding cassette transporter subfamily G member 1 (ABCG1), some of which are overexpressed on cancer cells such as rectal, brain, and metastatic prostate cancer [58]. However, some limitations must be resolved before clinical use of lipoproteins, including poor production scalability due to difficulty in their separation from co-isolated particles such as extracellular vesicles (EVs), batch-to-batch inconsistency, and the lack of cost-efficient manufacturing methods [57]. Recent advances in recombinant protein production may address these challenges by enabling controlled expression in host systems (such as bacterial or yeast platforms), improving batch reproducibility, facilitating large-scale production under standardised conditions, and simplifying purification processes to reduce contamination with EVs and other biological components.

Ferritin (Ft) is an endogenous iron storage protein present across all organisms [59]. The protein shell is a spherical 24-mer nanocage apoferritin (AFt) composed of heavy (H; 21 kDa) and light (L; 19 kDa) chain subunits. AFt has an outer diameter of ~12 nm and an internal cavity of ~8 nm. Ft features eight 3-fold (hydrophilic) and six 4-fold (hydrophobic) channels, which allow Fe^2+^ entry and H^+^ transport, respectively, and can be leveraged to encapsulate cargo agents via the nanoreactor (diffusion) route (e.g., temozolomide (TMZ) [60,61]. pH (or urea)-induced dis/re-assembly of the AFt provides a facile method for encapsulation of larger cargo (e.g., QDs) [51,62]. For example, iron oxide nanoparticles encapsulated in Ft, magnetoferritin, have been successfully explored for tumour imaging through their peroxidase-like activity [63]. AFt binds to cells via TfR1 (H-chain recognition), ref. [64], scavenger receptor class A member of 5 (SCARA5, L-chain recognition), and chemokine (C-X-C motif) receptor 4 (CXCR4, H- or L-chain recognition) [65]. Due to the high iron demand and proliferative nature of cancer cells, TfR1 is upregulated across multiple cancer types [27,66], enabling targeted cargo delivery to tumours [67]. Another Ft-like nanocage, DNA-binding proteins from starved cells (Dps), comprising 12 subunits, has ~9 nm outer diameter and a ~4.5 nm inner cavity and also offers a biocompatible platform for cargo delivery [68]. The hollow structure and pH-responsive self-assembly of Ft and Dps proteins make them highly attractive nanoplatforms for receptor-targeted cancer theranostics.

Immunogenic viral protein capsids, also known as virus-like particles (VLPs), also hold considerable promise due to their self-assembling properties. Protein-based natural nanocarriers that lack viral genetic material are non-infectious, biocompatible, and biodegradable [69], with uniform morphology, nanoscale dimensions (~20–200 nm), and chemical or genetic functionalisation opportunities [69]. Commonly used VLPs are derived from animal viruses such as hepatitis B virus (HBV) and human papillomavirus (HPV), bacteriophages such as emesvirus zinderi (MS2) and oubevirus durum (Qβ), and plant viruses including cowpea mosaic virus and tobacco mosaic virus (TMV) [70]. Their hollow interiors have been used to load cargoes, such as chemotherapeutics [71], siRNA [72], and imaging agents (QDs [73], gadolinium (Gd)-based agent [74]), by pH- or temperature-induced self-assembly. It is important to note that VLPs can stimulate the immune system by mimicking infectious pathogens (which limits treatment to the initial dose); thus, they have also been widely explored as platforms for novel nanovaccines for cancer [75].

Of fibrin-type proteins, collagen is the main component of the extracellular matrix and has low immunogenicity, which makes it attractive for theranostic formulations [40]. Desolvation, emulsification-solvent evaporation, electrospraying, and microfluidics can be used for the production of collagen-based formulations [40,76]. Collagen hydrolyses into amphoteric gelatin, which contains both anionic and cationic groups, as well as hydrophobic groups [77]. Therefore, gelatin offers a high density of accessible functional groups for attachment of targeting ligands and imaging moieties [78]. Silk fibroin (the primary structural protein in silk) from Bombyx mori (the domestic silk moth) is used in oncology [79]; it offers opportunities for chemical or genetic functionalisation (e.g., RGD peptides [80,81], folic acid [82,83], and monoclonal antibodies [84]), enabling both EPR-based passive targeting and receptor-mediated active targeting [85]. Silk fibroin consists of glycine, alanine, and serine, with hydrophobic and hydrophilic domains alternating along the chain, which are critical for its structural stability and functional versatility [40]. Desolvation, salting-out, and electrospraying methods have been successfully used for silk protein-based nanoparticle production [86]. Despite the advantages of silk protein, clinical translation is currently limited by batch-to-batch inconsistency, short shelf life, and complex functionalisation [86]. These limitations may be mitigated through recombinant protein production methods which may enhance the translational potential of silk-based theranostic platforms.

The advantageous properties of proteins for targeted cargo delivery have been widely investigated, with multiple reports of encapsulation of either therapeutic [60,87,88,89,90] or imaging [51,91] agents, with recent growing interest in theranostics [92,93,94].

## 4. Imaging Modalities for Protein-Based Theranostics

Imaging modalities play a key role in early cancer diagnosis and detection, guiding treatment planning and surgical interventions [95]. In protein-based theranostics, imaging agents for CT, MRI, PET, SPECT, and NIR imaging have been investigated [96] as well as hybrid/multi-modal imaging approaches [93,97].

**Figure 2:**
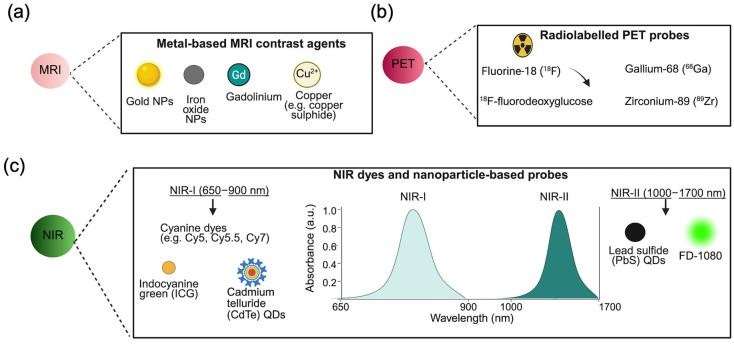
Representative imaging probes are used in protein-based theranostic platforms. (**a**) Metal-based magnetic resonance imaging (MRI) contrast agents, including gold nanoparticles (NPs), iron oxide NPs, gadolinium-based, and copper-based agents (e.g., copper sulphide). (**b**) Radiolabelled positron emission tomography (PET) probes, such as fluorine-18 (^18^F), ^18^F-fluorodeoxyglucose (^18^F-FDG), gallium-68 (^68^Ga) and zirconium-89 (^89^Zr). (**c**) Near-infrared (NIR) dyes and nanoparticle-based probes, including cyanine dyes (e.g., Cy5, Cy5.5, Cy7), indocyanine green (ICG), cadmium telluride quantum dots (CdTe QDs) for NIR-I (650–900 nm), lead sulphide quantum dots (PbS QDs), and small molecule dye FD-1080 for NIR-II (1000–17,000 nm). Created with BioRender.

While imaging capabilities offer high resolution and accuracy, there are often concerns associated with the use of contrast agents. CT is used in early investigations of cancers; however, allergies to contrast agents are possible [98]. GdIII-based contrast agents (T1 contrast agents) and iron oxide NPs (T2 contrast agents) (Figure 2a) are used in MRI to distinguish tumour grades and tumour-related responses [99]. Gd-based agents (gadodiamide, gadopentetate, dimeglumine, and gadoversetamide) have been withdrawn from clinical use because of safety concerns, such as the risk of nephrogenic systemic fibrosis, in patients with advanced chronic kidney disease [100]. This is associated with the release of toxic Gd^3+^ ions from less stable chelates via transmetallation with endogenous metal ions such as Zn^2+^ or Cu^2+^, leading to tissue deposition and progressive fibrosis [100]. Additional concerns regarding gadolinium have been reported; for example, repeated administration of gadodiamide has been shown to result in gadolinium accumulation in rat brain tissue, likely through binding to macromolecules [101]. PET and SPECT can visualise metabolic processes using radiotracers, providing high sensitivity imaging modalities used for tumour staging, treatment response assessment, recurrence detection as well as whole body assessment [98]. Fluorine-18 (^18^F) and gallium-68 (^68^Ga)-labelled folate derived conjugates have been used as PET imaging agents targeting the FR, as have glucose metabolism tracers (e.g., ^18^F-fluorodeoxyglucose) and amino acid transport tracers (Figure 2b) [8]. Protein-based nanocarriers (e.g., AFt) have been explored as PET radiotracers by radiolabelling with zirconium-89 (89Zr), enabling TfR1-targeted tracking of theranostic protein nanocarriers [102].

Among imaging modalities, fluorescence imaging offers real-time imaging with high contrast and resolution as well as reduced scattering, absorption and tissue autofluorescence [103]. Imaging in the near-infrared region (NIR) has gained attention owing to its sensitivity and harmless radiation. NIR-I (650–900 nm) commonly employs dyes such as indocyanine green (ICG; C_43_H_47_N_2_NaO_6_S_2_), cyanine 7 (Cy7; C_41_H_48_BF_4_N_3_O_4_)/cyanine 5.5 (Cy5.5; C_40_H_43_CIN_2_O_2_), and Alexa Fluor 680/750 (C_39_H47BrN_4_O_13_S_3_) (Figure 2c) [104]. However, NIR-I is limited by depth penetration and resolution in tumour imaging. These limitations can be resolved by NIR-II (1000–1700 nm) imaging using small molecule NIR-II dyes (e.g., FD-1080) or inorganic emitters such as QDs (Figure 2c) [105]. Importantly, QDs offer promise as NIR-II probes for deep tissue applications. Currently, fluorescence image-guided surgery (FGS) helps surgeons to visualise tumours in real time and facilitate improved resection of tumour tissue; for example, 5-aminolevulinic acid (5-ALA) is Food and Drug Administration (FDA)-approved for GBM via protoporphyrin IX (PpIX) fluorescence [106].

## 5. Advanced Preclinical Models for Assessment of Theranostic Agents

Traditional in vitro two-dimensional (2D) models are most frequently used in theranostics’ research. However, they present limitations, lacking important features of tumours in vivo, including the ability to reproduce structural and biochemical tumour complexity, gradients of oxygen and nutrients, heterogenous receptor expression, and cell–cell and cell–extracellular matrix (ECM) interactions [107]. Such limitations create a translational gap in the evaluation of theranostic formulations. More advanced preclinical models such as scaffold-free methods (e.g., spheroids), scaffold-based three-dimensional (3D) cultures, organoids, patient-derived models, ex vivo tumour slices, and in vivo studies have been widely used [108,109,110]. These models enable assessment of tumour penetration, biodistribution, therapeutic response and imaging abilities of theranostic platforms prior to in vivo studies, thus reducing animal use in accordance with the 3Rs framework for ethical animal research replacement, reduction, and refinement, and streamlining clinical translation [111]. Each model presents distinct advantages and disadvantages.

Three dimensional spheroids provide a practical advanced model for theranostic applications to assess depth penetration, cytotoxicity, and the combination of therapeutic and diagnostic activity. Spheroids, aggregates of one or multiple cell types, can be generated by scaffold-free and scaffold-based approaches [112]. Scaffold-free models are simpler than scaffold-based techniques; however, they outperform 2D monolayers by reproducing gradients in nutrients, oxygen, and pH and by forming hypoxic and necrotic cores observed in the tumours. Although they remain well studied for investigating theranostic applications and their sequential treatments, they have some limitations in terms of the lack of immune components and ECM. In contrast, scaffold-based spheroids (hydrogels, collagen, and matrigel) better reflect cell-cell and cell-matrix interactions, offering a more relevant environment to in vivo ECM [112,113].

Patient-derived models (patient-derived organoids or patient-derived explants) present genetic and molecular heterogenous models of the original tumours, mimicking in vivo tumour microenvironments, and are amenable to high resolution imaging [114,115]. Alternatively, organ-on-a-chip (microfluidic) systems and bioprinting (3D/4D) technologies enable the generation of physiologically relevant 3D cultures [116]. These advanced models recapitulate organ structure and function via controlled fluidics and better emulate dynamic processes such as delivery of nutrients, drugs, and imaging agents but require the use of specialised devices [117]. These advanced models are valuable for difficult to reach tumours (e.g., GBM), where delivery barriers (e.g., BBB) limit therapeutic and imaging performance [115]. Although numerous intermediate advanced models, such as spheroids [108] and organoids [109], have been developed to quantify nanoparticle penetration, induced cytotoxicity, and imaging abilities, ex vivo and in vivo studies [110,118] remain the most widely used platforms for protein-based theranostic applications.

## 6. Protein-Based Theranostic Applications in Oncology

Protein-based theranostic platforms have been developed that have demonstrated promising responses in cancer models. Table 1 summarises the protein-based theranostic studies for combined imaging and therapy, detailing the protein platform, targeting receptor, cargo, imaging modality, and experimental model. Although most studies remain at the preclinical stage, they provide proof-of-concept for image-guided and tumour-selective treatments.

Among those, albumin is attractive for translational theranostics since it is already used clinically [56] as a drug nanocarrier and can be functionalised to incorporate imaging agents. For example, Zayed et al. developed a mannose-modified BSA-coated cadmium telluride (CdTe) QD theranostic platform with an overall diameter of 193.9 ± 4.8 nm by co-encapsulating resveratrol, a natural polyphenol and phytoalexin with anti-cancer activity, and the hydrophilic chemotherapy drug pemetrexed. This work demonstrated in vivo both fluorescence imaging capabilities of breast cancer and significant (*p* < 0.05) tumour growth reduction in MCF-7 and MDA-MB-231 breast cancer models [2]. Mannose-targeted theranostic formulations showed synergy (combination index (CI) < 1) and lowered IC_50_ in both cell lines compared to free resveratrol and pemetrexed combinations and non-targeted BSA nanoparticles. The inclusion of CdTe QDs further decreased IC_50_ values compared to QD-free BSA NPs, which was attributed to the generation of reactive oxygen species (ROS) leading to apoptosis. The strong fluorescence of the QDs (excitation wavelength: 450 nm, emission wavelength: 530 nm) enabled imaging of tumour cells in vitro and in vivo in an Ehrlich ascites mammary tumour-bearing mouse model.

**Figure 3:**
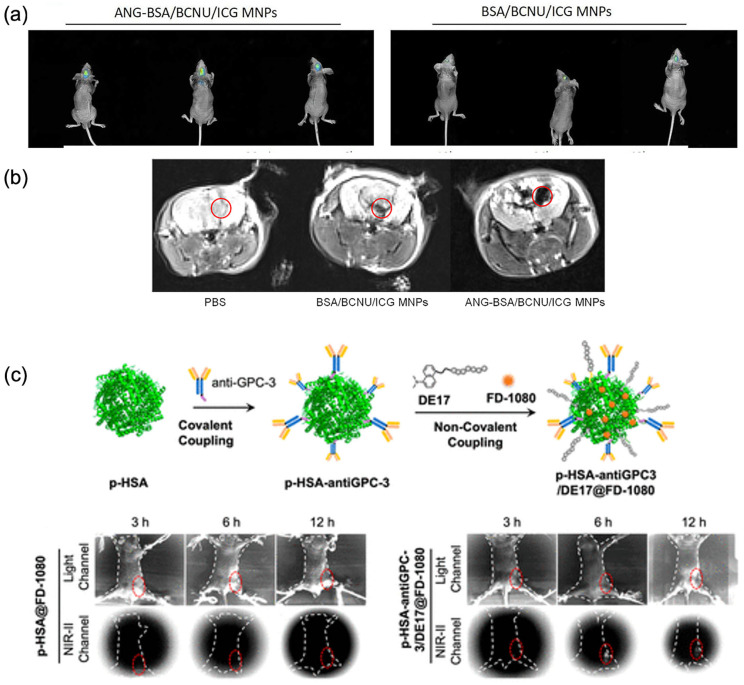
(**a**) In vivo comparison of angiopep-2 functionalised bovine serum albumin (BSA)-coated with superparamagnetic iron oxide, co-loaded with carmustine and indocyanine green (ICG) (termed ANG-BSA/BCNU/ICG MNPs) non-functionalised BSA (termed BSA/BCNU/ICG MNPs). (**b**) In vivo MRI comparison of ANG-BSA/BCNU/ICG MNPs and BSA/BCNU/ICG MNPs. Red circles indicate the targeting capabilities in the brain region following administration. Reproduced from Ref. [119]. (**c**) The preparation of functionalised human serum albumin (HSA) nanoparticles and their in vivo near-infrared II (NIR-II) fluorescence imaging in HepG2-bearing tumour mice. Reproduced from Ref. [120]. In the figure, orange circles represent FD-1080, a NIR-II imaging agent. Red cycles highlight the fluorescence intensity changes following administration of modified HSA nanoparticles, indicating alterations at specified time points.

Targeting of brain tumours has also been reported with theranostic agents based on angiopep-2-functionalised BSA-coated superparamagnetic iron oxide NPs co-loaded with carmustine and ICG (85 ± 10 nm diameter). These agents were targeted to lipoprotein receptor-related protein (LRP) overexpressed in pericytes and smooth muscle cells of the BBB as well as GBM 293T and U87MG cells [119], providing contrast in both MRI and in vivo fluorescence imaging. Fluorescence signals from the nanoparticles were detected from 30 min post-injection, with maximum intensity after 12 h, and were still detectable up to 48 h. Angiopep-2-functionalised BSA NPs accumulated within the brain tumour, confirming efficient targeting (Figure 3a,b, Table 1). Advantages of the imaging in NIR-II were demonstrated by Dou et al. with engineered HSA-based NPs modified by covalent cysteine conjugation and self-assembly, incorporation the NIR-II dye FD-1080 [120]. This dual modification enabled specific targeting of glypican-3, a biomarker of hepatocellular carcinoma, and imaging NIR-II imaging in mice bearing hepatocellular carcinoma cell-derived xenograft tumours showed high fluorescence intensity confirming theranostic agent accumulation in HepG2-derived xenograft tumours (Figure 3c, Table 1).

Targeted delivery of PTX and iron oxide nanocrystals (diameter 11 nm) was reported with folate-functionalised lipoprotein-mimetic NPs to FR-positive MCF-7 cells (compared to FR-negative A549 cells). In vivo, these theranostic agents demonstrated MRI signal enhancement in breast cancer xenograft models [121]. Reconstituted HDLs (rHDLs) were engineered by covalently attaching bifunctional chelators to cholesterol on their surface, allowing radiolabelling for SPECT/PET imaging while encapsulating therapeutic agents in their lipophilic interior; rHDLs retained Scavenger Receptor Type B1-mediated tumour targeting, demonstrating their potential as a personalised lipoprotein-based theranostic platform [122].

The unique structure of Ft nanocages (diameter ~12 nm) provides a nanoplatform for integration of drug molecules, MRI and fluorescent imaging agents into a single construct for targeted cancer theranostics. Veroniaina et al. co-encapsulated manganese dioxide (MnO_2_) and doxorubicin (DOX) within recombinant human H-Ft, demonstrating combined MRI signalling ability and growth inhibitory activity in cervical (HeLa), ovarian (SKOV3), and breast (4T1) cancer cells [123]. In the study, exploiting Mn^2+^ as a T1-shortening MRI agent, a bright T1-weighted signal was observed in the tumour region only 20 min post-injection, alongside a reduction in malignant cell proliferation (Table 1). In another study, Nasrollahi et al. encapsulated graphene oxide QDs, iron and DOX into Ft protein cages [93]. The fluorescence and MRI imaging abilities, attributed to graphene oxide QDs and iron content, respectively, were demonstrated in the 2D triple-negative breast cancer cell model, the MDA-MB-231, confirming its potential as a multifunctional platform for breast cancer. The intrinsic imaging capabilities of human L-ferritin and drug-loaded magnetoferritin have been demonstrated for MRI-based applications [3,124]. For instance, Bitonto et al. investigated L-ferritin as a theranostic agent in TS/A breast cancer cells in vitro and in an orthotopic breast cancer mouse model in vivo [124].

**Figure 4:**
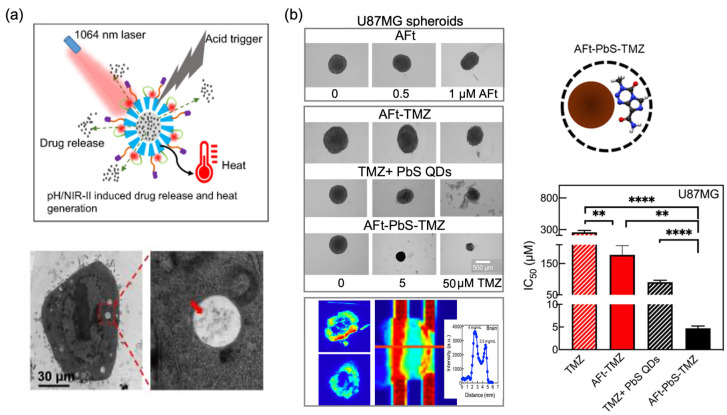
(**a**) Schematic illustration of folic acid-functionalised apoferritin theranostic formulation co-loaded with paclitaxel (PTX) and IR-1061 for pH/ NIR-II-induced drug release and heat generation and its accumulation in endosomes or lysosomes after incubation for 3 h in the 4T1 breast cancer model. Red arrow indicates cellular accumulation of the theranostic apoferritin formulation via receptor-mediated endocytosis. Reproduced from Ref. [126]. (**b**) In vitro growth inhibition studies of horse spleen AFt co-loaded with a lead sulphide quantum dot (PbS QD) and temozolomide (TMZ) in 3D U87MG spheroids, together with their NIR-II imaging in GBM spheroids and healthy brain tissue. Reproduced from Ref. [94]. (**) represents *p* < 0.01, and (****) represents *p* < 0.0001.

Horse spleen AFt is the most widely used the Ft family protein adopted for the development of theranostic agents. For instance, Cutrin et al. entrapped drug molecules and imaging agents together into AFt nanocages for the first time, encapsulating curcumin and MRI contrast agent (Gd-HPDO3A) [125]. These AFt nanocages were designed to target hepatocytes via the SCARA5 receptor, aiming to prevent hepatocellular damage in toxic-induced acute hepatitis while also enabling evaluation of drug delivery efficiency by MRI. Recently, PTX and IR1061 (NIR-II organic dye) were co-encapsulated within folic acid-functionalised AFt nanocages (IR-AFN@PTX-FA) for tumour-targeted and pH/NIR-II triggered synergistic photothermal chemotherapy (Figure 4a). Evaluation in 4T1 breast cancer models, both in vitro and in vivo, was carried out (Table 1) [126]. The NPs were internalised via both clathrin- and caveolae-mediated endocytosis and subsequently localised within lysosomes (Figure 4a). Additionally, compared to free PTX, IR-AFN@PTX-FA displayed prolonged blood circulation and enabled targeted delivery to breast tumours via folic acid functionalisation of AFt; biodistribution peaked at 24 h post-injection, with predominant accumulation in the liver and spleen. Similarly, DOX and the NIR fluorescent dye ADS-780 were co-encapsulated into AFt nanocages, and their activity was evaluated for photothermal therapy (PTT) in colorectal cancer models (Table 1) [127]. In vivo studies using BALB/c nude mice bearing HT-29 tumours showed therapeutic activity without any significant toxic side effects over a 27-day period. More recently, the theranostic potential of AFt nanocages was investigated by co-encapsulating a single lead sulphide quantum dot (PbS QD) together with >300 TMZ molecules within horse spleen AFt nanocages, termed AFt-PbS-TMZ, demonstrating the targeted theranostic potential of this protein-based platform against intractable brain tumours using scaffold-free U87MG glioblastoma spheroid models (Table 1) [94]. These AFt-based theranostic NPs (AFt-PbS-TMZ) exploit TfR1-mediated enhanced delivery to GBM cells and were able to: (i) overcome TMZ resistance via AFt encapsulation (rapidly downregulating O6 methylguanine-DNA methyltransferase) [94]; (ii) enhance TMZ activity compared to naked TMZ; (iii) generate a strong NIR-II signal in ~400 µm U87MG spheroids due to the presence of PbS QD within the AFt cavity (Figure 4b). In one study, copper and the ring-opened TMZ triazene 5-(3-methyl-1-triazenyl)imidazole-4-carboxamide (MTIC) were co-encapsulated in horse spleen AFt [128]; AFt encapsulation led to prolonged MTIC half-life. In a separate study, copper phenanthroline and TMZ were co-encapsulated in the same protein nanocarrier [129]. In both cases, enhanced growth inhibition in glioma cells was reported. Collectively, these studies demonstrate potential for future applications incorporating co-encapsulation within AFt cages of copper-based theranostic agents. More broadly relevant to Ft-based theranostics, Jin et al. reported on a bioengineered Ft-based nanocarrier (tHFn(+)) enabling targeted siRNA delivery against GBM and using Cy5.5-labelled HFn/HFn(+) in orthotopic mouse models, demonstrating BBB penetration and tumour accumulation by NIR imaging [130].

VLPs have been used for cancer theranostics, including PTT and MRI imaging [131]. SPARC-targeted M13 bacteriophage VLPs were conjugated with DOX and Alexa Fluor 488 and were subsequently evaluated in C42B (SPARC-positive) and DU145 (which express lower levels of SPARC), prostate cancer cells (Table 1) [71]. The researchers also developed SPARC-targeted M13 bacteriophage VLPs for magnetic iron oxide NPs, which generated T2 (dark-contrast) MRI signals for tumour detection in vivo in prostate cancer xenograft models derived from C4-2B and DU145 cells in immunodeficient mice (Table 1) [132]. In another study, bacteriophage Qβ-based VLPs were developed following the attachment of gold NPs (6.2 ± 0.2 nm), serving as X-ray or PET contrast agents, while >500 DOX molecules were loaded into icosahedral capsids via diffusion through 1.3–1.5 nm diameter threefold axis pores of Qβ (Table 1) [133]. This multifunctional platform suggested cytotoxic activity against macrophages and lung cancer cells in vitro, with minimal morphological alterations observed in the irradiated area. However, these in vitro investigations require further validation to assess their tumour-targeting, biodistribution, and therapeutic efficacy.

**Figure 5:**
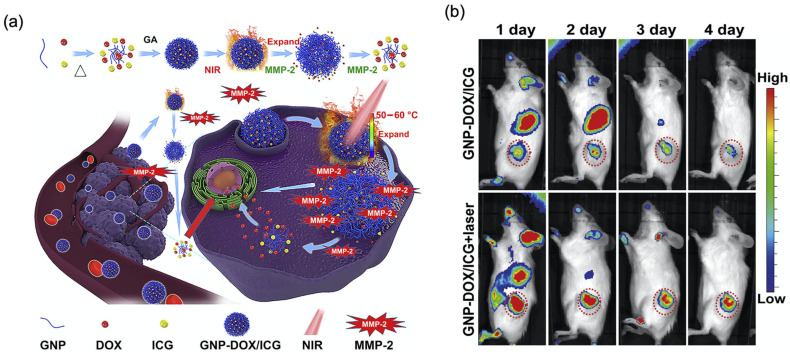
(**a**) Schematic representation of preparation of a gelatin-based theranostic formulation containing doxorubicin (DOX) and indocyanine green (ICG), and subsequent blood circulation, cellular uptake, and drug release enabled by laser irradiation and matrix metalloproteinase-2 (MMP-2) degradation. (**b**) In vivo tumour accumulation of GNP-DOX/ICG with and without laser irradiation at 1, 2, 3, and 4 days in 4T1 tumour-bearing mice. Reproduced from Ref. [4].

Collagen-based nanocarriers have been investigated for in vivo fluorescence and MRI tracking as well as for anti-tumour activity, building on their favourable biocompatibility and biodegradability, as described above. For instance, Xia et al. developed folate-functionalised gelatin NPs (particle size: 180 ± 15 nm, PDI < 0.1) prepared using a microfluidic device, encapsulating CuS NPs (15 nm), Fe3O4 NPs (12 ± 3 nm), and curcumin (Table 1) [134]. The targeting ability of this multifunctional platform was evaluated in lung adenocarcinoma A549 cells (low FR expression) and breast adenocarcinoma MCF-7 cells (high FR expression). Cells were treated with folate-functionalised gelatin NPs for 1 h, 4 h, 8 h, and 12 h to evaluate time-dependent uptake. The results showed higher uptake in MCF-7 cells compared to A549 cells, together with potential for multimodal theranostic use: Fe_3_O_4_ enabling MRI contrast, CuS enabling photothermal conversion, and curcumin serving as the therapeutic agent. In another study with gelatin-based NPs, a photothermal agent ICG, and DOX were co-loaded into gelatin NPs (GNP-DOX/ICG) to generate a platform against breast cancer (Figure 5a, Table 1) [4]. ICG provided NIR fluorescence for in vivo imaging and photothermal guidance in 4T1-bearing mice (Figure 5b), highlighting the potential of gelatin-based theranostic systems. Ex vivo and in vivo fluorescence imaging indicated that GNP-DOX/ICG exhibited prolonged circulation, attributed to gelatin. The highest tumour-associated fluorescence signal was observed at 24 h post-injection, with additional accumulation reported in the liver, spleen, lung, and kidney.

**Figure 6:**
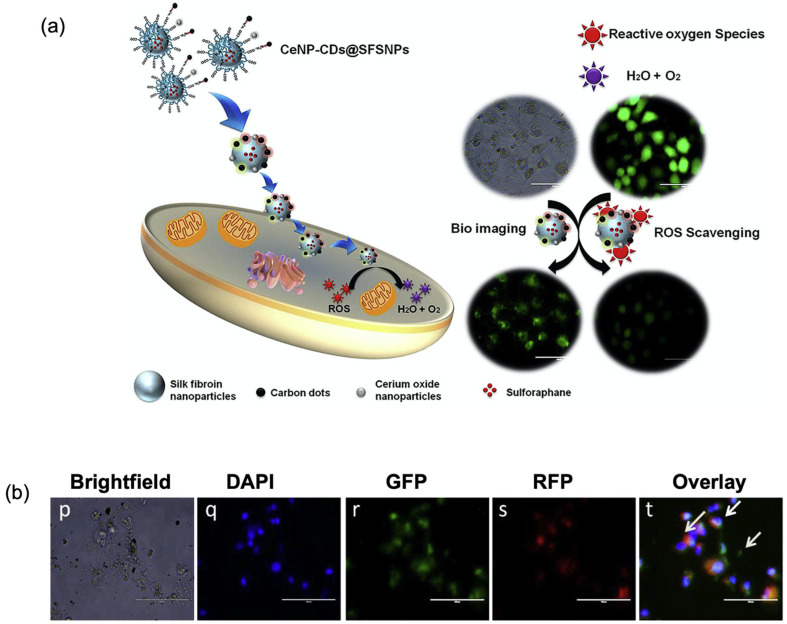
(**a**) Schematic representation of a silk fibroin-based multifunctional nanocomposite (CeNP-CD@SFSNPs) and (**b**) fluorescence images of A549 cells 24 h after treatment with nanocomposite, demonstrating intracellular distribution of carbon dots (CD) (scale bar =100 μm). Panels p–t show different staining channels: p (brightfield), q (4′,6-diamidino-2-phenylindole (DAPI, nuclei)), r (green fluorescence protein (GFP)), s (red fluorescent protein (RFP)), and t (overlay of all channels). White arrows show cytoplasmic localisation. Reproduced from Ref. [92].

Silk proteins represent one of the engineerable protein platforms that could be harnessed for targeted cancer theranostics. In one study, bioengineered spider silk was functionalised with adhesion peptides, including metal-binding peptides and HER2-binding peptides, and was used to co-deliver magnetic iron oxide NPs and DOX to HER2-positive cancer cells (Table 1) [135]. Scanning electron microscopy (SEM) images revealed that these composites had a spherical morphology. Upon exposure to an external magnetic field, magnetic NPs generated heat, leading to cancer cell apoptosis. The platform was evaluated in SKBR3 human breast cancer cells, which overexpress HER2. In another example, Passi et al. designed a multifunctional platform based on silk fibroin nanocarriers derived from Bombyx mori loaded with cerium oxide NPs, carbon QDs, and antioxidant drug sulforaphane using a desolvation method (Figure 6a) [92]. The resulting spherical protein nanocarriers (particle size: 69.0 ± 26.4 nm) were designed to deliver the drug to combat oxidative stress while enabling intracellular imaging of A549 lung cancer cells (Figure 6b). The study is limited by the lack of targeted surface functionalisation as well as the absence of in vivo biodistribution, stability, and imaging efficacy. However, the researchers reported that further studies on surface functionalisation are ongoing to achieve targeted theranostic applications. Furthermore, Gao et al. exploited the co-encapsulation capability of microfluidic-assisted silk fibroin NPs from Bombyx mori to load both epirubicin (EPI) and copper sulphide (CuS), achieving synergistic, targeted photodynamic- and photothermal therapy (PDT/PTT), combined with chemotherapy in in vitro breast cancer models [136]. Under 808 nm NIR irradiation, FR-targeted silk fibroin NPs (378 nm diameter) showed enhanced cytotoxicity against breast cancer cells (MDA-MB-231 and MCF-7). This enhanced activity was associated with EPI release (55% release rate at pH 5 without NIR irradiation; 85% release rate at pH 5 with NIR irradiation) and an increase in alkyl radicals, which was related to the release of copper ions in the acidic microenvironment, while maintaining low toxicity toward non-cancerous HEK-293 cells. The researchers concluded that future work should focus on patient-derived samples, advanced 3D tumour models (e.g., spheroids, organoids), and in vivo experiments. In addition, this protein-based nanocarrier system holds a promise as a theranostic platform owing to the presence of CuS.

**Table 1:** Protein-based theranostic nanoparticles (NPs) developed for cancer imaging and treatment.

Protein/ Modification	NP Size	Target/ Receptor	Therapeutic Cargo	Imaging Agent/ Modality	Cancer Type	Model	Ref.
Mannose-modified BSA	193.9 ± 4.8 nm	Mannose receptor	Resveratrol (encapsulation efficiency (EE)%: 68.1 ± 5.1%) Pemetrexed (conjugation efficiency%: 50.9 ± 1.9%)	CdTe QD/fluorescence imaging	Breast	In vitro and in vivo, the Ehrlich ascites mammary tumour-bearing mouse model	[2]
Angiopep-2 functionalised BSA	85 ± 10 nm	LRP	Carmustine (EE%: 15%)	ICG, superparamagnetic iron oxide/MRI and in vivo fluorescence imaging	Brain	In vitro *(*293T and U87MG cells) and in vivo orthotopic GBM nude mice	[119]
Glycipan-3-targeted peptide-modified HSA	~220 nm	Glycipan-3	-	NIR-II dye FD-1080/NIR-II fluorescence imaging	Hepatocellular	In vivo (xenograft model)	[120]
Recombinant human H-Ft	12.05 ± 1.3 nm	TfR1	DOX (encapsulation rate%: 48.4%)	MnO_2_/MRI imaging	Cervical, ovarian, breast	In vitro heLa, SKOV3, and 4T1 cancer cells	[123]
Ft	13.5 ± 1.0 nm	TfR1	DOX (loading capacity: 35%)	Graphene oxide QDs iron complex/fluorescence and MRI imaging	Breast	In vitro MDA-MB-231 cells	[93]
Folic acid-functionalised AFt	35 nm	FR, TfR1	PTX (loading ratio 145.6%)	IR1061/NIR-II fluorescence imaging	Breast	4T1 models both in vitro and in vivo (subcutaneous xenograft model)	[126]
AFt	60.1 nm	TfR1	DOX (loading efficiency: 48%)	NIR dye ADS-780/NIR-I fluorescence imaging	Colorectal	BALB/c nude mice bearing HT-29 tumours	[127]
Horse spleen AFt	12.1 ± 0.6 nm	TfR1	TMZ (>300 molecules per AFt, EE%: 74.4 ± 11.2%)	PbS QD/NIR-II fluorescence imaging	GBM	In vitro 2D and 3D U87MG spheroids	[94]
M13 bacteriophage VLPs	-	SPARC	DOX (257 DOX molecules per VLP)	Alexa Fluor 488/fluorescence imaging	Prostate	In vitro 2D C42B and DU145 cells	[71]
M13 bacteriophage VLPs	~181.4 nm	SPARC	-	Magnetic iron oxide NPs/MRI imaging	Prostate	In vivo xenograft model	[132]
Qβ-based VLPs	64.83 ± 0.219 nm	-	DOX (up to 500 molecules per VLP)	Gold NPs/fluorescence imaging	Lung	In vitro 2D A549 cells	[133]
Gelatin	180 ± 15 nm	FR	Curcumin	CuS NPs Fe_3_O_4_ NPs/fluorescence imaging	Lung, breast	In vitro 2D A549 and MCF-7 cells	[134]
Gelatin	Stimuli-responsive size changes (71.6 nm → 160.8 nm → 33.2 nm)	-	DOX	ICG/fluorescence imaging	Breast	Ex vivo and 4T1 tumour in vivo	[4]
Silk spider	-	HER2	DOX	Magnetic iron oxide NPs/MRI imaging	Breast	In vitro SKBR3 cells	[135]

## 7. Conclusions

Protein-based nanocarriers play an advantageous role in cancer theranostics by enabling selective targeting either through inherent receptor interactions or via functionalisation with antibodies, ligands, or peptides that bind to overexpressed receptors in many cancers, such as EGFR, TfR1, and FR. Given tumour heterogeneity and related challenges (drug resistance and recurrence) in oncology, integrating therapeutic and imaging agents into a single platform allows real-time pharmacodynamic (PD) confirmation of target engagement, early detection of treatment response, and adaptive dosing. However, closing the translational gap between preclinical and clinical studies will require advanced preclinical models (e.g., 3D spheroids, organoids, organ-on-chip) and in vivo PK/PD and efficacy evaluation before human trials. When coupled with biomarker-guided patient selection, targeted protein-based theranostics may provide a path towards personalised medicine.

Despite promising preclinical results, clinical translation of protein-based theranostics is limited by tumour heterogeneity, variable intratumoural penetration, carrier-specific immunogenicity, regulatory barriers, cost, and scale-up limitations. Preclinical and clinical validation is required to establish imaging accuracy, therapeutic activity and efficacy, and targeting specificity. In addition, comprehensive safety assessments, including detailed toxicity studies for both protein-based nanocarriers and imaging agents, are required. In terms of biocompatibility concerns related to protein-based nanocarriers, particularly those derived from foreign sources (e.g., bacteriophages, spider silk proteins, horse spleen, or BSA), potential immunogenicity remains a critical consideration. Although many protein carriers are generally regarded as biocompatible, non-human proteins may trigger innate or adaptive immune responses. The formation of anti-drug antibodies may alter PK, reduce therapeutic activity, and accelerate systemic clearance. In contrast, human-derived proteins may present lower immunogenic risk compared to foreign ones. Therefore, carrier selection, recombinant production and engineering strategies are required to minimise immune responses and facilitate clinical translation. Regarding NP production, manufacturing and scale-up present reproducibility and quality-control challenges. Microfluidic systems are promising, but they still require further validation [87]. To address these limitations, several strategies can be employed to improve the commercial feasibility of protein-based theranostics, including the use of recombinant expression systems (e.g., bacteria and yeast) to reduce production costs, standardised protein scaffolds to simplify manufacturing, and advanced purification technologies to ensure quality and scalability. Protein-based nanocarriers hold potential for future personalised medicine through integration with patients’ genetic and molecular profiles (e.g., receptor expression profile). All-in-one platforms that co-deliver diagnostics and therapeutics may disrupt multiple oncogenic pathways, overcome resistance, and improve management of intractable tumours. The combination of machine learning/artificial intelligence (AI) technology, proteomics, and molecular biology can guide formulation design and optimisation, and safety assessment can be guided to develop more effective and safer therapies that minimise side effects combat drug resistance mechanisms, thus speeding the translation process and ultimately improving patients’ quality of life.

## Data Availability

No new data were created or analyzed in this study.

## Abbreviations

The following abbreviations are used in this manuscript:

2D	Two-dimensional
3D	Three-dimensional
ABCG1	Adenosine triphosphate-binding cassette transporter subfamily G member 1
AFt	Apoferritin
BBB	Blood–brain barrier
BSA	Bovine serum albumin
CT	Computed tomography
CXCR4	Chemokine (C-X-C motif) receptor 4
Cy5.5	Cyanine 5.5
Cy7	Cyanine 7
DNA	Deoxyribonucleic acid
DOX	Doxorubicin
ECM	Extracellular matrix
EGFR	Epidermal growth factor receptor
EPI	Epirubicin
FR	Folate receptor
Ft	Ferritin
GBM	Glioblastoma
gp60	Glycoprotein 60
HBV	Hepatitis B virus
HER2	Human epidermal growth factor receptor 2
HPV	Human papillomavirus
HSA	Human serum albumin
ICG	Indocyanine green
LDLR	Low density lipoprotein receptor
LRP	Lipoprotein receptor-related protein
MRI	Magnetic resonance imaging
MS2	Emesvirus zinderi
MTIC	5-(3-methyl-1-triazenyl)imidazole-4-carboxamide
NIR	Near-infrared imaging
NPs	Nanoparticles
NSCLC	Non-small cell lung cancer
OVA	Ovalbumin
PbS QD	Lead sulphide quantum dot
PD	Pharmacodynamic
PDT/PTT	Photodynamic and photothermal therapy
PET	Positron emission tomography
PTX	Paclitaxel
QDs	Quantum dots
Qβ	Oubevirus durum
RTK	Receptor tyrosine kinase
SCARA5	Scavenger receptor class A member 5
SEM	Scanning electron microscopy
SPARC	Secreted protein acidic and rich in cysteine
SPECT	Single-photon emission computed tomography
SRB1	Scavenger receptor class B type 1
TfR1	Transferrin receptor 1
TMV	Tobacco mosaic virus
TMZ	Temozolomide
VLPs	Virus-like particles
